# An integrated characterization of *Picea abies* industrial bark regarding chemical composition, thermal properties and polar extracts activity

**DOI:** 10.1371/journal.pone.0208270

**Published:** 2018-11-27

**Authors:** Duarte M. Neiva, Solange Araújo, Jorge Gominho, Angélica de Cássia Carneiro, Helena Pereira

**Affiliations:** 1 Centro de Estudos Florestais, Instituto Superior de Agronomia, Universidade de Lisboa, Portugal; 2 Universidade Federal de Viçosa (UFV), Minas Gerais, Brazil; College of Agricultural Sciences, UNITED STATES

## Abstract

The present work determines the chemical and thermal characteristics as well as the phytochemical and antioxidant potential of the polar extractives of the *Picea abies* bark from an industrial mill, their wood and bark components and also different bark fractions obtained by mechanical fractionation (fine B_1_, Φ<0.180 mm, medium B_3_, 0.450 < Φ<0.850 mm and coarse B_6_, 2 < Φ<10 mm). The aim is to increase the knowledge on the *Picea abies* bark to better determine possible uses other than burning for energy production and to test an initial size reduction process to achieve fractions with different characteristics. Compared to wood, bark presented similar lignin (27%), higher mineral (3.9% vs 0.4%) and extractives (20.3% vs 3.8%) and lower polysaccharides (48% vs 71%) contents. Regarding bark fractions the fines showed higher ash (6.3%), extractives (25%) and lignin (29%) than the coarse fraction (3.9%, 19% and 25% respectively). Polysaccharide contents increased with particle size of the bark fractions (38% vs 52% for B_1_ and B_6_) but showed the same relative composition. The phytochemical profile of ethanol and water extracts presented higher contents for bark than wood of total phenols (2x higher), flavonoids (3x higher) and tannins (4-10x higher) with an increasing tendency with particle size. Bark antioxidant activity was higher than that of wood for ferric-reducing antioxidant power (FRAP, 10 vs 6 mmolFe^2+^/g_Ext_ for the ethanol extract) and free radical scavenging activity (DPPH, 6 vs 18 mg/L IC50 for the ethanol extract) methods. The different bark fractions antioxidant activity was very similar. Bark thermal properties showed a much lower volatiles to fixed carbon ratio (V/FC) than wood (3.1 vs 5.2) although the same higher heating value (20.3 MJ/kg). The fractions were quite similar. Bark presented chemical features that point to their possible upgrade, whether by taking advantage of the high extractives with bioactive compounds or the production potential for hemicellulose-derived oligomers with possible use in nutraceutical and pharmaceutical industries.

## Introduction

Norway spruce (*Picea abies* (L.) Karst.) is a major softwood species in Europe used extensively in pulp mills for mechanical and kraft pulp production, and in sawmills to produce wood components. The industrial processing originates large quantities of biomass as residual materials that accumulate at mill site and may be considered as side-streams available for valorization under a full resource utilization concept.

Barks are non-wood forest products that are receiving increased attention as a potential feedstock for an integrated valorization—the so-called bark-based biorefinery—given their availability and concentration at industrial processing sites, as well as their potential for a combination of value-added applications *e*.*g*. chemicals and bio-products, that add to their current use as solid fuel [1,2]. Barks of the different industrial tree species show great diversity in structural and chemical composition, as well as in physical and mechanical fractionation properties, thereby leading to specific valorization routes. Numerous bark characterization studies have been recently carried out in view of their potential use in biorefineries [3–9].

Norway spruce bark is one of the barks that has been researched quite intensively, since it is an abundant material i.e. it represents 10–15% volumetric content in logs, and is still an undervalued feedstock because it is used mostly for fuel in combustion, although its rich chemical composition allows considering a better valorization as a source of high-value chemicals e.g. directed to production of adhesives, resins and plastics as well as to bioactive extracts [10].

The chemical composition of Norway spruce bark has been reported for the bark as a whole [11, 12] as well as for the inner and outer bark fractions [13]. Norway spruce bark is characterized by a large proportion of extractives e.g. 21.6% [14] or 28.3% [15]. The hydrophylic extractives have attracted attention, namely tannins [16, 17] and stilbenes [18] as well as lipophilic extractives [19].

Polysaccharides are also major constituents of Norway spruce e.g. together, hemicelluloses, pectins and cellulose constitute about 50% of the inner bark, 33% of the outer bark [13] and 40% of the whole bark collected after debarking in a pulp mill [20, 14].

In this sense, a bark biorefinery that first extracts valuable compounds such as extractives [21, 22], tannins [10, 23], non-cellulosic polysaccharides [20, 24] and cellulose [25], and thereafter converts the residue into biofuels and energy could be interesting [26].

The industrial barks accumulated at mill yards have additional specific features that result from the harvesting, handling and debarking processes that may affect the feedstock composition *e*.*g*. presence of wood material, mineral and extraneous contaminations. For instance, Ngueho Yemele [27] observed a high wood content of 19.9% in the industrial spruce bark from a sawmill. Since composition of wood and bark differ greatly, experimental results obtained with pure bark may not translate exactly into the industrial scale, therefore advising the characterization of the specific industrial bark.

The industrial bark of *Picea abies* collected at the pulp mill yard was studied here in view of its potential use as a source of chemicals within a bark-based biorefinery. The wood content in the industrial bark was determined and mechanical fractionation of the bark into different sized particles was made. The fractions were evaluated in relation to chemical features including summative chemical composition and the phenolic profile of ethanol and water extracts (total phenolic compounds, tannins and flavonoids) and their antioxidant activity, as well as thermal properties including proximate and ultimate analysis, thermogravimetric analysis (TGA) and derivative thermogravimetric analysis (DTG). The valorization routes of this industrial residual stream are discussed under the concept of a full resource use (zero waste philosophy) and as a potential biomass source within a biorefinery concept.

## Material and methods

### Sampling and fractionation

A 100 kg sample of large chips from Norway spruce (*Picea abies* (L.) Karst.) bark was collected after the debarking from a sawmill located in Jyvaskyla, Finland. The chips were air dried for several days under well ventilated conditions and frequent mixing, followed by oven drying at 40°C for 3 days.

An aliquot of the industrial bark was visually observed and the wood (W) chips were separated from the bark (B) fractions and quantified. The bark fraction was knife-milled with a Retsch SM 2000 mill to pass a 10x10 mm screen (B) and sieved to six mesh sizes fractions: B_1_ (mesh <80, Φ <0.180 mm); B_2_ (mesh 60/80, 0.180<Φ<0.250 mm); B_3_ (mesh 40/60, 0.250<Φ<0.450 mm); B_4_ (mesh 20/40, 0.450<Φ<0.850 mm); B_5_ (mesh >20, 0.850<Φ<2 mm);B_6_ (2<Φ<10 mm). Each fraction was weighed and the yield calculated as o.d. mass.

The chemical analysis were performed on the samples B_1_ (fine), B_3_ (medium) and B_6_ (coarse), as well as on the unsieved B sample that represents the bark as a whole, and the wood (W). The combination of the wood and the bark fractions in their respective proportions represent the whole industrial bark as obtained.

Before analysis, the samples B_6_ and B were milled to pass a 1 mm ouput sieve and the entire material obtained was used. The wood sample was milled and the 40/60 mesh fraction (0.250<Φ<0.450mm) used for analysis.

### Chemical analysis

Ash content was determined by TAPPI standard T15 os-58 and extractives by successive Soxhlet extraction with dichloromethane, ethanol and water overnight for each solvent. Total lignin was determined in the extractive-free material as acid insoluble lignin (klason) and soluble lignin according to TAPPI standards T222 om-88 and UM250 om-83 respectively. Klason lignin was adjusted taking in consideration its ash content. Hydrolysis liquor resulting from T222 om-88 was used to ascertain polysaccharides composition regarding neutral monosaccharides, glucuronic acid, galacturonic acid and acetates by separation through a Dionex ICS-3000 High Pressure Ion Chromatographer, using an Aminotrap plus Carbopac SA10 column. All the chemical analysis were made in triplicate.

The ash composition was determined as follows: Cl by EN 15289:2011 standard; B by spectrophotometry (420 nm) after oven burning at 600°C; the remaining elements were determined after nitro-perchloric acid digestion followed by atomic absorption spectroscopy (Ca, Mg, Fe, Zn, Cu, Mn, Ni, Pb, Cr), spectrophotometry (P and S at 725 and 420 nm respectively) and emission flame photometry (K). Two duplicates were tested and result expressed as average.

### Ethanol and water extractives

The composition and antioxidant activity of the ethanol and water extracts obtained by successive extraction were analyzed in relation total phenols (TPC), flavonoids (FC) and condensed tannins (CTC) contents as previously described [28]. TPC results were reported as mg gallic acid equivalents (GAE)/g_Extract_ and FC and CTC as (+)-catechin equivalents (CE)/g_Extract_ through a calibration curves.

The antioxidant activity of the extracts was estimated by two methods: ferric-reducing antioxidant power (FRAP), expressed in mmol Fe(II)/g extract and the results compared to standards (ascorbic acid, catechin and gallic acid); free radical scavenging activity (DPPH) as described by Sánchez-Moreno [29]. The DPPH results were expressed as IC_50_ (extract concentration required for 50% DPPH inhibition) and as antioxidant activity index (AAI = final concentration of DPPH in the control sample/IC_50_) which takes in consideration the mass of DPPH and test sample decreasing the concentration influence of the DPPH solution used [30]. The antioxidant activity is classified as poor if AAI<0.5, moderate if 0.5<AAI<1, strong 1<AAI<2 and very strong when AAI>2. DPPH results were compared to a natural and a synthetic standards ((+)-catechin and trolox, respectively.

All the analyses were made in triplicate and results expressed as average with standard deviation.

### Thermal properties

Proximate analysis was determined according to ASTM E870-82 standard for ash and volatile matter, with fixed carbon calculated by difference to 100%. Ultimate analysis followed ASTM D5373-08 standard using a Perkin-Elmer II 2400 elemental analyzer (Shelton, CT, USA), with oxygen content determined by difference after C, H, N, and ash content determination. The higher heating value (HHV) was determined following ABNT NBR 8633 standard using an adiabatic bomb calorimeter IKA300 (Staufen, Germany).

Thermogravimetric analysis (TGA) and derivative thermogravimetry (DTG) were carried out in a Shimadzu DTG-60H (Kyoto, Japan) with dynamic nitrogen atmosphere (gas flow of 50 mL min^-1^) between 10–680°C with a 10°C min^-1^ heating rate, using 2 mg ± 0.1 mg samples in a platinum container.

## Results

### Fractionation

The industrial bark included wood pieces (W) that could be clearly out singled and corresponded to 15.9% (m/m) of the total.

Mechanical grinding and granulometric sieving of the bark-only material (B) contained in the industrial bark yielded the following fraction distribution: B_1_ (< 0.180 mm) 3.5%, B_2_ (0.18<Φ<0.25 mm) 1.9%, B_3_ (0.25<Φ<0.45 mm) 4.0%, B_4_ (0.45<Φ<0.85 mm) 9.6%, B_5_ (0.85<Φ<2.00 mm) 27.6% and B_6_ (2.00<Φ<10.00 mm) 53.4%. The material was brittle and fractionated easily into coarse granules (B_5_ and B_6_) that together constituted 81.0% of the total.

### Chemical composition

Table 1 summarizes the results obtained for the chemical composition of the industrial bark and of its wood and bark fractions, regarding content of ash, extractives and lignin as well as the monomeric composition of polysaccharides (neutral sugars, uronic acids and acetates).

**Table 1:** Chemical composition (in % o.d. mass) of the industrial bark (IB) of *Picea abies*, the wood (W) and bark-only (B) fractions, as well as of three granulometric fractions obtained after grinding and sieving of bark (B_1_, < 0.180 mm, B_3_ (0.25<Φ<0.45 mm) and B_6_ (2.00<Φ<10.00 mm).

	IB	W	B	B1	B3	B6
**Ash**	**3.32 ± 0.07**	**0.38 ± 0.01**	**3.88 ± 0.08**	**6.28 ± 0.11**	**4.21 ± 0.10**	**3.87 ± 0.02**
**Extractives**	**17.64 ± 1.30**	**3.78 ± 0.21**	**20.25 ± 1.5**	**25.18 ± 0.19**	**21.69 ± 0.63**	**18.74 ± 0.18**
Dichloromethane	4.68 ± 0.05	0.81 ± 0.03	5.41 ± 0.05	8.17 ± 0.24	6.44 ± 0.06	4.81 ± 0.06
Ethanol	3.93 ± 0.24	0.87 ± 0.05	4.51 ± 0.27	6.62 ± 0.37	5.37 ± 0.16	4.13 ± 0.23
Water	9.03 ± 1.16	2.11 ± 0.20	10.34 ± 1.34	10.39 ± 0.56	9.88 ± 0.63	9.80 ± 0.12
**Lignin**	**26.92 ± 0.74**	**27.22 ± 0.29**	**26.86 ± 0.82**	**28.75 ± 0.92**	**29.92 ± 1.59**	**24.81 ± 0.30**
Klason	26.08 ± 0.70	26.9 ± 0.30	25.93 ± 0.77	27.74 ± 0.96	29.03 ± 1.63	23.84 ± 0.31
Acid soluble	0.83 ± 0.06	0.32 ± 0.01	0.93 ± 0.07	1.01 ± 0.04	0.89 ± 0.05	0.98 ± 0.04
**Polysaccharides**	**51.56 ± 0.92**	**71.18 ± 1.51**	**47.87 ± 0.81**	**37.86 ± 0.32**	**42.61 ± 1.49**	**52.27 ± 0.26**
Rhamnose	0.48 ± 0.02	0.07 ± 0.00	0.55 ± 0.03	0.56 ± 0.01	0.49 ± 0.05	0.73 ± 0.04
Arabinose	4.56 ± 0.34	1.32 ± 0.02	5.17 ± 0.4	4.05 ± 0.20	4.15 ± 0.35	6.31 ± 0.13
Galactose	1.96 ± 0.09	2.03 ± 0.03	1.95 ± 0.1	1.74 ± 0.03	17.7 ± 0.07	2.17 ± 0.09
Glucose	29.49 ± 0.21	43.89 ± 0.73	26.78 ± 0.11	20.71 ± 0.23	23.97 ± 1.31	29.39 ± 0.2
Xylose	4.97 ± 0.31	7.11 ± 0.94	4.57 ± 0.19	3.18 ± 0.05	4.22 ± 0.49	4.21 ± 0.08
Mannose	4.42 ± 0.35	14.34 ± 1.80	2.55 ± 0.08	2.54 ± 0.01	2.95 ± 0.06	2.27 ± 0.03
Galacturonic acid	4.48 ± 0.12	0.74 ± 0.01	5.62 ± 0.14	4.42 ± 0.07	4.36 ± 0.17	6.54 ± 0.10
Glucuronic acid	0.22 ± 0.01	0.09 ± 0.00	0.25 ± 0.01	0.28 ± 0.01	0.25 ± 0.02	0.24 ± 0.01
Acetic acid	0.62 ± 0.01	1.60 ± 0.06	0.44 ± 0.00	0.38 ± 0.02	0.45 ± 0.02	0.42 ± 0.01
**Total**	**99.4 ± 0.4**	**102.5 ± 1.2**	**98.9 ± 0.3**	**98.1 ± 0.6**	**98.4 ± 1.1**	**99.7 ± 0.7**

The bark (B) has a high content of extractives (20.3% of o.d. material) mainly constituted by polar compounds soluble in ethanol and water, that represent together 73% of the total extractives. Lignin content is 26.9% and polysaccharides represent 47.9% of the bark. Polysaccharides are mainly constituted by glucose (55.9% of the total units) while the composition of hemicelluloses is dominated by a high presence of arabinose and xylose (10.8% and 9.6% of the total units, respectively) and of galacturonic acid (11.7%); galactomannans are present in lower amounts with galactose and mannose representing respectively 4.1% and 5.3% of the total units.

The composition of wood (W) is different mainly regarding the much lower content in extractives that represent only 3.8% of the material and the also much lower ash content i.e. 0.38% vs. 3.88% in bark (Table 1). The polysaccharides, that represent 71.2% of the wood, have a composition that differs from that of bark by the dominance of galactomannans (mannose and galactose correspond to 20.2% and 2.9% of the total units), with less proportion of uronic acids and by a higher acetylation degree (acetic acid corresponds to 2.5% of the units vs. 0.9% in bark).

The composition of the industrial bark as a whole is similar to the bark composition with differences in direct relation to the proportion of the wood it contains (Table 1).

The three granulometric fractions show some chemical compositional differences. The fine fraction B_1_ has a higher ash content (6.3%) that decreases in the B_3_ and B_6_ fractions (4.2% and 3.9% respectively). The fine fraction also shows the highest content of extractives (25.2%), that decrease with the increase of particle size to 18.7% in the B_6_ fraction, mostly due to a decrease in the dichloromethane soluble compounds. The coarse fraction B_6_ differentiates from the other two by the lower proportion of Klason lignin (23.8% and 27.7% for B_6_ and B_1_ respectively) and the highest polysaccharide content (52.3% and 37.9% for B_6_ and B_1_ respectively) The monomeric composition of the polysaccharides was similar between fractions with a slight enrichment in glucose in the coarse fraction (56.2% and 54.7% of all units for B_6_ and B_1_ respectively).

### Ash mineral composition

Table 2 presents the results obtained for the ash mineral composition. Calcium is the most important mineral of bark, corresponding to 1.5% of the material, followed by potassium (0.2%); the content of manganese is comparatively high (0.05%). Cl and S are present corresponding to 0.04 and 0.05% respectively.

**Table 2:** Mineral composition (in % o.d. mass or ppm) of the industrial bark (IB) of *Picea abies*, the wood (W) and bark-only (B) fractions, as well as of three granulometric fractions obtained after grinding and sieving of bark (B_1_, < 0.180 mm, B_3_ (0.25<Φ<0.45 mm) and B_6_ (2.00<Φ<10.00 mm).

	IB	W	B	B1	B3	B6
Ca (%)	1.25	0.14	1.46	1.74	1.55	1.52
K (%)	0.21	0.14	0.22	0.25	0.24	0.24
P (%)	0.05	0.02	0.06	0.08	0.07	0.05
Mg (%)	0.07	0.02	0.08	0.09	0.07	0.08
S (%)	0.05	0.04	0.05	0.05	0.05	0.04
Cl (%)	0.04	0.03	0.04	0.04	0.04	0.02
Cu (ppm)	9	34	4	4	8	4
Fe (ppm)	96	53	104	253	228	54
Zn (ppm)	137	31	158	158	146	170
Mn (ppm)	397	73	458	436	394	486
B (ppm)	16	25	14	21	20	23
Ni (ppm)	1.6	0.9	1.8	2.2	3.1	1.0
Pb (ppm)	3.8	3.5	3.9	3.5	7.0	3.3
Cr (ppm)	2.5	1.6	2.7	4.3	6.0	2.5

In wood, the content of the different minerals is much lower given the overall low ash content. It is noteworthy that the potassium content has a much higher relative proportion than in bark.

The different granulometric fractions of bark show a quite similar composition with exception for the iron content which is four to five times lower in the coarse fraction regarding the medium and fine fractions (Table 2).

### Ethanol and water extractives

The phenolic composition of the ethanol and water extracts obtained by the successive extraction with both solvents is shown in Table 3 regarding contents in total phenolics, flavonoids and condensed tannins. The antioxidant activity of the extracts is also included as measured by the FRAP and DPPH methods.

**Table 3:** Chemical composition (total phenolics, flavonoids, condensed tannins) and antioxidant properties (FRAP and DPPH) of the ethanol and water extracts of the industrial bark (IB) of *Picea abies*, the wood (W) and bark-only (B) fractions, as well as of three granulometric fractions obtained after grinding and sieving of bark (B_1_, < 0.180 mm, B_3_ (0.25<Φ<0.45 mm) and B_6_ (2.00<Φ<10.00 mm). Determinations on standards are included for FRAP (ascorbic acid, catechin and gallic acid) and for DPHH (trolox and catechin) antioxidant properties.

	IB	W	B	B1	B3	B6	Standard
**Phenolics (mg GAE/gExt)**							
Ethanol	778 ± 33	391 ± 41	851 ± 32	710 ± 15	772 ± 22	849 ± 31	
Water	176 ± 9	119 ± 9	187 ± 9	311 ± 25	288 ± 14	193 ± 8	
**Flavonoids (mg CE/gExt)**							
Ethanol	424 ± 10	151 ± 16	476 ± 9	407 ± 1	407 ± 12	479 ± 11	
Water	88 ± 7	37 ± 4	98 ± 7	101 ± 4	109 ± 1	106 ± 7	
**Condensed tannins (mg CE/gExt)**							
Ethanol	309 ± 5	38 ± 4	360 ± 6	254 ± 7	271 ± 7	350 ± 18	
Water	35 ± 2	10 ± 1	40 ± 2	61 ± 1	56 ± 1	40 ± 2	
**FRAP antioxidant activity**							
Ethanol (mmolFe^2+^/gExt)	9.4 ± 0.2	6.0 ± 0.1	10.0 ± 0.0	9.0 ± 0.0	9.0 ± 0.0	10.0 ± 0.0	
Water (mmolFe^2+^/gExt)	2.4 ± 0.1	1.6 ± 0.1	2.6 ± 0.1	2.6 ± 0.1	2.8 ± 0.0	2.5 ± 0.1	
Ascorbic acid (mmolFe^2+^/g)							17 ± 0.4
Catechin (mmolFe^2+^/g)							18 ± 0.5
Gallic acid (mmolFe^2+^/g)							40 ± 1.0
**DPPH antioxidant activity**							
Ethanol IC50 (mg/L)	8.1 ± 0.4	17.8 ± 1.3	6.3 ± 0.2	5.1 ± 0.4	4. 7 ± 0.2	6.8 ± 0.3	
Ethanol AAI	3.3 ± 0.1	1.3 ± 0.1	3.7 ± 0.1	4.6 ± 0.3	5.0 ± 0.2	3.5 ± 0.2	
Water IC50 (mg/L)	23.9 ± 0.9	40.6 ± 2.5	20.7 ± 0.6	15.8 ± 0.6	13.7 ± 0.2	20.4 ± 1.2	
Water AAI	1.0 ± 0.0	0.6 ± 0.0	1.1 ± 0.0	1.5 ± 0.1	1.7 ± 0.0	1.1 ± 0.1	
Trolox IC50							3.5 ± 0.2
AAI							6.7 ± 0.3
Catechin IC50							2.7 ± 0.4
AAI							8.6 ± 0.7

The ethanol extracts contain much higher values of phenolics, flavonoids and tannins than the subsequent water extracts. The bark ethanol extracts have a high proportion of phenolic compounds (851 mg GAE/g_Ext_) in which flavonoids and condensed tannins constitute the major classes (476 mg CE/g_Ext_ and 360 mg CE/g_Ext_ respectively). The bark water extracts contain a much lower amount of phenolic compounds i.e. 187 mg GAE/g_Ext_ total phenolics, 98 mg CE/g_Ext_ flavonoids and 40 mg CE/g_Ext_ condensed tannins.

Compared to bark, wood has a lower phenolic content especially of tannins e.g. 391 mg GAE/g_Ext_ total phenolics, 151 mg CE/g_Ext_ flavonoids and 38 mg CE/g_Ext_ condensed tannins in the ethanol extracts (Table 3).

The extracts from the different bark granulometric fractions did not show considerable differences apart from the total phenolics in B_6_ that had a higher content in the ethanol extracts and a lower content in the water extracts.

The FRAP antioxidant activity of bark extracts (Table 3) showed that ethanol extracts have more antioxidant power than water extracts (10.0 mmolFe^2+^/g_Ext_ vs. 2.6 mmolFe^2+^/g_Ext_). The values are lower than those obtained for usual antioxidant compounds e.g. ascorbic acid, catechin or gallic acid (17, 18 and 40 mmolFe^2+^/g_Ext_ respectively). The wood had a lower antioxidant activity compared to bark.

As regards the free radical scavenging activity, the concentration of extracts required for 50% DPPH inhibition (IC50) was 6.3 and 20.7 mg/L for bark ethanol and water extracts respectively. Both extracts are less effective than trolox and catechin standards (IC50 = 3.5 and 2.7 mg/L respectively). The wood ethanol and water extractives have very low antioxidant activity (IC50 = 17.8 and 40.6 mg/L respectively). The bark ethanol extracts have an antioxidant activity index (AAI) above 2 (AAI = 3.3) that corresponds to a strong antioxidant classification.

The FRAP antioxidant activity of the different bark granulometric fractions is similar. The DDPH free radical scavenging activity of both ethanol and water extracts was similar for the three granulometric fractions with the coarser fraction always presenting a lower antioxidant activity than the other fraction.

### Thermal properties

Table 4 presents the results of the proximate and ultimate composition as well as the higher heating value. In comparison to wood, bark has more ash, less volatiles and more fixed carbon leading to a lower V/FC ratio (3.1 vs. 5.2). The elemental composition of bark and wood is similar and the differences of the H/C and O/C ratios are of small magnitude (1.39 *vs*. 1.49 and 0.63 *vs*. 0.67, respectively for bark and wood). The higher heating value (HHV) of bark and wood is the same at 20.3 MJ/kg. The bark fractions show small differences in thermal properties with the fixed carbon increasing from smaller to bigger fraction (22.5 vs 24 for B_1_ and B_6_ respectively). The coarser fraction showed a lower higher heating value than the other two fractions.

**Table 4:** Proximate and ultimate analysis (in % o.d. mass) and high heating value (MJ/kg) of the industrial bark (IB) of *Picea abies*, the wood (W) and bark-only (B) fractions, as well as of three granulometric fractions obtained after grinding and sieving of bark (B_1_, < 0.180 mm, B_3_ (0.25<Φ<0.45 mm) and B_6_ (2.00<Φ<10.00 mm).

	IB	W	B	B1	B3	B6
**Proximate analysis**						
Volatiles (%)	74.7 ± 0.6	83.6 ± 0.4	73.0 ± 0.7	71.7 ± 0.3	72.1 ± 0.9	72.3 ± 0.5
Fixed carbon (%)	22.2 ± 0.6	16.1 ± 0.4	23.4 ± 0.6	22.5 ± 0.6	23.6 ± 0.9	24.0 ± 0.4
Ashes (%)	3.1 ± 0.1	0.3 ± 0.0	3.6 ± 0.1	5.8 ± 0.2	4.3 ± 0.0	3.7 ± 0.0
V/FC ratio	3.4 ± 0.0	5.2 ± 0.1	3.1 ± 0.1	3.2 ± 0.1	3.1 ± 0.1	3.0 ± 0.1
**Ultimate analysis**						
C (%)	48.6	49.1	48.5	49.0	50.0	48.6
H (%)	5.7	6.1	5.6	5.7	5.8	5.8
N (%)	1.0	0.7	1.0	1.2	1.0	1.0
S (%)	0	0	0	0	0	0
O (%)	41.4	43.7	41.0	37.8	39.0	40.7
Atomic H/C ratio	1.40	1.49	1.39	1.40	1.39	1.43
Atomic O/C ratio	0.64	0.67	0.63	0.58	0.58	0.63
**HHV (MJ/kg)**	20.3	20.3	20.3	20.6	20.7	20.1

**Fig 1:**
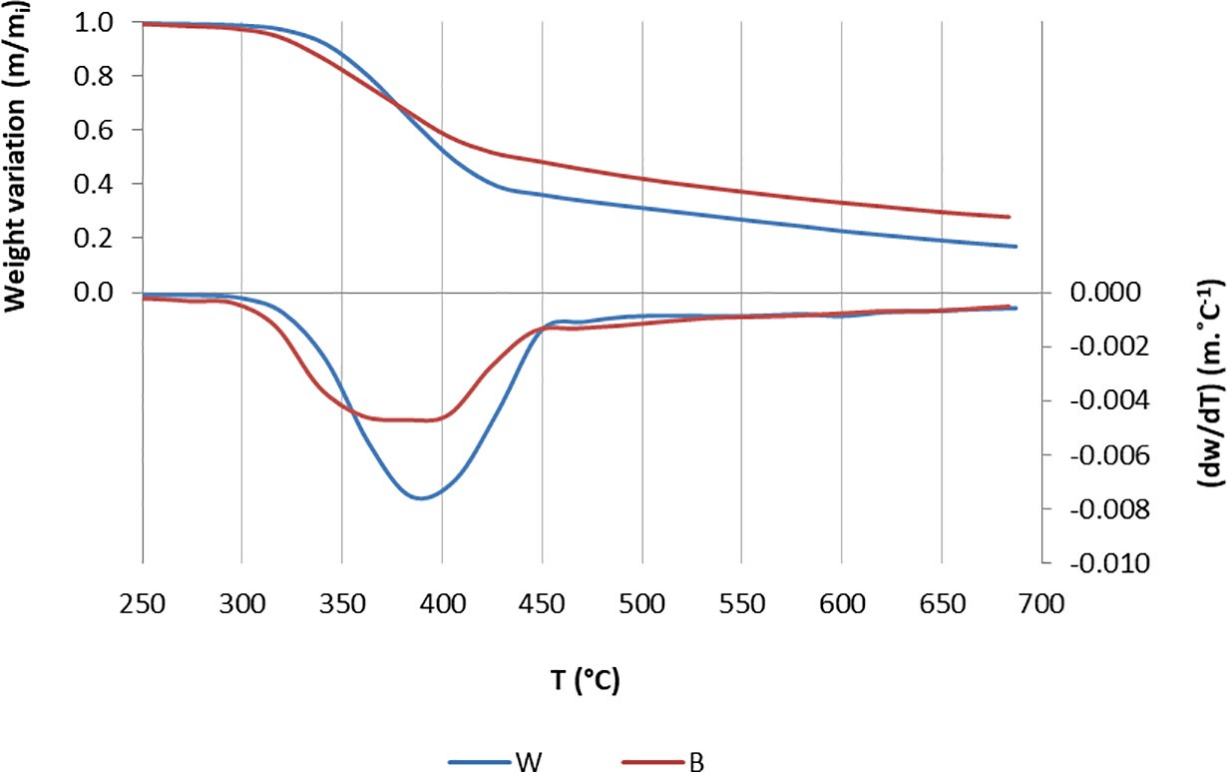
TGA and DTG of the wood (W) and bark-only (B) fractions of *Picea abie*s industrial bark.

Fig 1 shows the results for the TGA and DTG analysis made between 250 and 690°C. Mass loss starts near 300°C but the thermal degradation at higher temperatures differs somewhat between bark and wood. For bark the highest rate of mass loss spreads in the temperature range of 360–403° and for wood the highest degradation rate is reached at 385°C. After 450°C, both wood and barks show a constant rate of degradation of 0.001 mg°C ^-1^.

The bark fractions do not show differences in thermal degradation and the TGA and DTG curves are largely superposed.

## Discussion

The industrial bark of *Picea abies* collected at mill site included a significant proportion of wood that derived from the debarking process. A high wood content in a sawmill residual bark was previously reported at up to 21% [10, 27]. Although this wood proportion represents a loss in the wood supply to the subsequent main process, it is important to notice that Norway spruce bark has structural features that are incompatible with some wood applications, namely those of pulp and veneer production. The technical relevance of such contamination to the process depends on the structural features of the specific bark; in some cases, the impact may not be so important e.g. for fibrous barks such as those of *Eucalyptus globulus* that may even be considered as a pulping feedstock [6, 31, 32]. However, this is not the case of *Picea abies* bark and a conservative option is taken by the mills to avoid bark contamination in the feedstock. Therefore, if the industrial *Picea abies* bark stream is directed for specific targeted applications, the presence of wood in amounts similar to those found in this work–ca. 16%—has to be taken into consideration. This means that the composition of the industrial bark will somewhat differ from that of the bark itself (Table 1).

The mechanical fractionation of *Picea abies* bark showed that the material is easily comminuted and fines are not produced in significant amounts e.g. particles < 0.25 mm represented only 5.4% of the material. This is of practical importance for raw-material pre-processing and shows that sieving operations may not be required if they are not detrimental to the following unit operations. Similar results on the fractionation behavior of Norway spruce bark were also reported by Miranda [14].

The *Picea abies* bark contains a substantial amount of extractives, mainly polar compounds (14.8% of the bark, Table 1). Such high content of extractives has been reported in many studies with values similar to those found here [10, 13, 33, 34]. The high extractives content (20%), and substantial apolar fraction (5%) follows the same behavior than other softwood species, with Douglas-fir, loblolly pine, scots pine and stone pine showing values in the range of 19–30% total extractives and 2–7% apolar ones.[3, 14, 35, 36]

When targeting a specific industrial bark stream, the extractives content will depend on its wood proportion since wood has a much lower extractive content (3.0% of polar extractives, Table 1). When considering the extraction of polar compounds successively with ethanol and water, the results showed that most of the phenolics were extracted by ethanol (Table 3). In fact, the ethanol extracts contained a very high proportion of phenolic compounds, mostly constituted by flavonoids (476 mg CE/g_Ext_) and condensed tannins (360 mg CE/g_Ext_). The subsequent water extraction solubilizes the remaining phenolic compounds which correspond to much lower proportions of the extract (Table 3); in the water extract an important proportion of solubilized sugars should be present [10]. However, to fully account for the potential production of phenolic extracts from the *Picea abies* bark, the fraction extracted by water should also be taken into consideration given their yield in terms of the initial bark material e.g. ethanol only extracts about 65% of the total phenolics (Table 3). However, from a practical point of view, it should be considered that the ethanol and water extracts differ in relation to their concentration in phenolics (high concentration in the ethanol extract and lower in the subsequent water extract) and to their antioxidant activity.

The bark ethanol extracts are classified as being very strong antioxidants with an AAI above 3.5, although their free radical scavenging activity is lower than that of usual antioxidants, either synthetic or natural, e.g. trolox and catechin (6.7 and 8.6 respectively). The same applies to the FRAP antioxidant power which is half of that of usual antioxidant compounds e.g. ascorbic acid (10 mmolFe^2+^/g_Ext_ vs. 17 mmolFe^2+^/g_Ext_ respectively). Being a sequential extraction it is normal that the water extracts have lower antioxidant properties but nevertheless they still have compounds with antioxidant capability that are not solubilized in ethanol; since the water extractives yield is much higher than that of ethanol (Table 1), this fraction cannot be ignored if the aim is obtain compounds with antioxidant activity. When compared to wood, bark has a much higher extraction yield (five times more) and the compounds extracted also have higher antioxidant activity.

The high content of extractives in Norway spruce has attracted the attention of research and several studies showed their potential [37, 21]. The practical use of the bark for production of tannins was proposed by Kempainnen [10] who could obtain water extracts with up to 50% tannin content at pilot-scale. Lacoste [38] showed that purified tannins from *Picea abies* bark could be used to produce foams.

The composition of *Picea abies* bark regarding structural components (Table 1) is in line with published reports for this species [10, 14]. In comparison to other softwoods barks such as Douglas-fir, loblolly or Scots pines It shows lower lignin content (27% vs 30–44%) but higher polysaccharides (48% vs 24–38%) [3, 14, 35]. It is noteworthy that the bark hemicelluloses contain a high proportion of arabinose, xylose and galacturonic acid, together representing 32.1% of the polysaccharides while the composition of wood polysaccharides is quite different, with glucose representing 61.7% of the total, and mannose and galactose 23.1% (Table 1). The high hemicellulosic content of *Picea abies* bark allows to consider it as a potential source of oligosaccharides by mild treatments such as hydrothermal processes after the removal of polar extractives. Hydrothermal treatments have been applied to various types of biomass and proposed for production of xylooligosaccharides using e.g. rice straw [39], corn cobs [40] or even wood [41]. A further material valorization along chemical fractionation routes may consider delignification of the cellulose and lignin enriched solids to yield a cellulose-rich solid fraction and a soluble lignin [4, 25].

Regarding the compositional profile of the different granulometric fractions (Table 1), the fines showed a higher extractive content (approximately one third more extractives than the coarse fraction) as well as more mineral content. This enrichment in extractives and minerals in the smallest particles obtained after grinding was already found for *Picea abies* bark [14] as well as for barks of other species [7, 8] and for other biomass types [42,43]. As regards structural components, fines were enriched in lignin when reported on an extractive- and ash-free basis (42.0% vs. 32.0% in the coarse fraction), but the polysaccharide profile was similar, as also previously reported [14].

When compared to wood, bark had higher fixed carbon content which is also visible in the behavior of the TGA plot, leading to a higher weight variation after the 450°C. Bark starts to decompose at lower temperatures, probably due to the higher extractives content that are easily degraded. On the other hand, the DTG curve is less pronounced than for wood since bark has a lower volatiles content. The TGA and DTG plots of the three fractions were almost identical, which means that the chemical variations found (Table 1) did not alter significantly their thermal degradation in the absence of oxygen.

The results obtained point out that *Picea abies* industrial bark is suitable as a biomass source for valorization within a biorefinery concept, allowing fractionation into chemicals or an energy use. It should also be referred that the bark may be used for energy production either directly after collection (HHV of bark and wood is the same at 20.3 MJ/kg, Table 4) or at any point along the fractionation. Francezon and Stevanovic presented small decreases (up to 5%) on higher heating values of black spruce (*Picea mariana*) bark after several extraction processes have been reported [44]. The energetic content of the P*icea abies* bark is on the lower end of the range published for several softwood species that go from 19.6 MJ/kg up to 25 MJ/kg [45].

**Fig 2:**
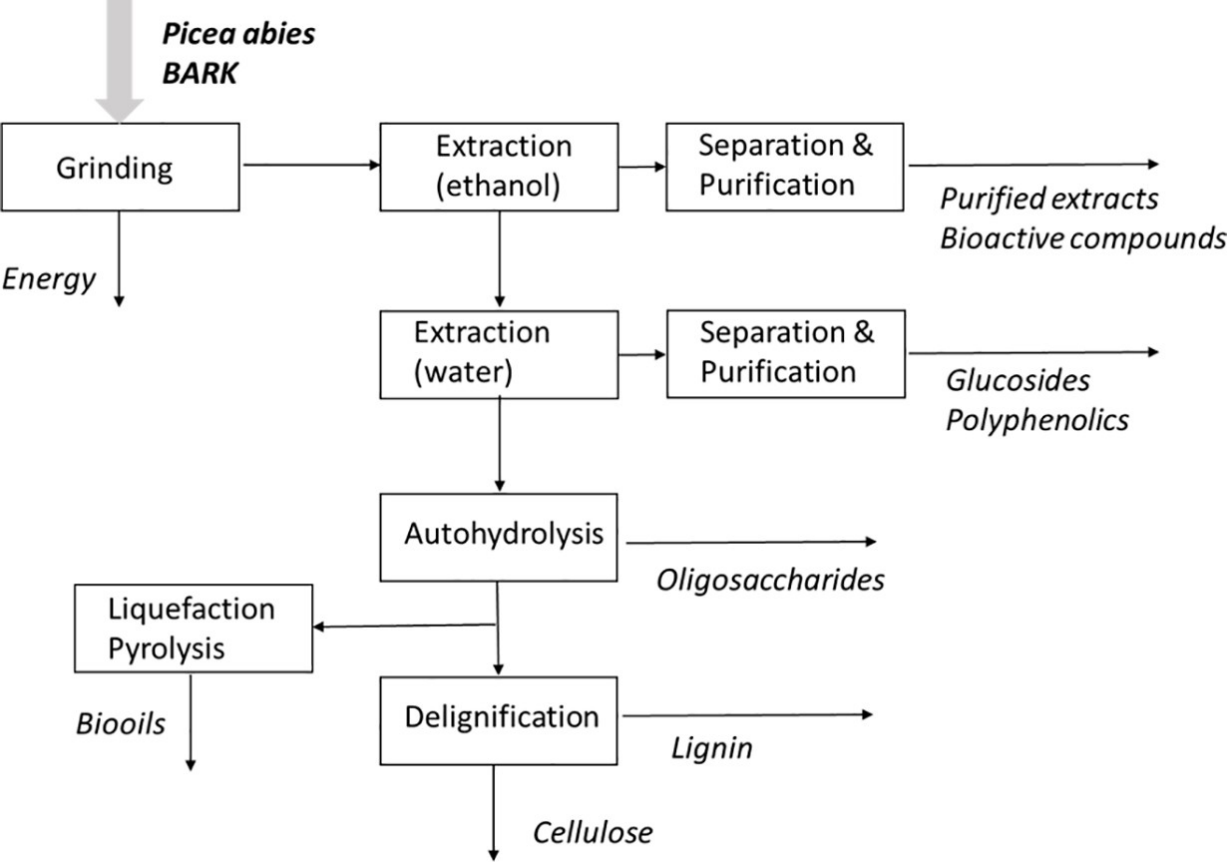
Example of possible fractionation sequence for Picea abies industrial bark biorefinery.

An example of a possible fractionation sequence is shown in Fig 2. Alongside the conventional use of bark as a solid biofuel, the *Picea abies* bark granulated material may be extracted by a solvent sequence and the obtained raw extracts purified to obtain bioactive compounds or chemicals, while the extracted solids may be either used for energy e.g. as biooils through liquefaction or pyrolysis, or used for carbohydrate oligomer production by e.g. hydrothermal treatments, and further delignified to yield lignin fractions and cellulose that can be directed to cellulose-based materials or bioethanol.

## Conclusions

The industrial bark has a substantial amount of wood and extraneous material derived from harvesting, handling and debarking processes leading to specific features that have to be accounted for if this material is to be used in a biorefinery context.

Chemically, bark is very different from wood, presenting a much higher extractive content, with the crude polar fraction showing very good antioxidant activity, and has similar lignin and lower polysaccharides content. The polysaccharides are richer in hemicelluloses, especially in arabinose and galacturonic acid as opposing the mannose in the wood. Regarding energy properties, bark presented a lower volatiles to fixed carbon ratio and a more controlled thermal degradation than wood but the same calorific value.

As for the fractionation of the bark in different sized particles, the fractions had some chemical differences (the fine fraction was richer in ash, extractives and lignin, and lower in polysaccharides contents) but were very similar in thermal characteristics. The mechanical fractionation as a first processing step in a biorefinery seems therefore unnecessary due to its operational costs.

Overall, the chemical and thermal characteristics of *Picea abies* bark show a possible upgrade potential, whether by taking advantage of the high content in extractives with bioactive compounds or the high amount of hemicelluloses allowing production of oligomers for possible use in nutraceutical and pharmaceutical applications or production of biomaterials such as biofilms or adhesives, while the remaining solid residue may be used for biofuels, chemicals or direct energy production.
